# A Cell-Penetrating Peptide and Signal Sequence Fusion DNA Vaccine Enhances CD8+ T Cell-Mediated Anti-Tumor Immunity Targeting HPV-16 Cervical Cancer Antigens

**DOI:** 10.26502/ogr0205

**Published:** 2026-07-17

**Authors:** Yu-Cheng Chang, Ky McSweeney, Yichu Xu, Yining Liu, Ya-Chea Tsai, T.-C. Wu, Chien-Fu Hung

**Affiliations:** 1 Department of Pathology, Johns Hopkins University School of Medicine, 1550 Orleans Street, CRB II 307, Baltimore, MD, 21287, USA; 2 Department of Oncology, Johns Hopkins University School of Medicine, MD, USA; 3 Department of Molecular and Cell Biology, Johns Hopkins University, Baltimore, MD, USA.; 4 Department of Obstetrics and Gynecology, Johns Hopkins University School of Medicine, Baltimore, MD, USA; 5 Department of Molecular Microbiology and Immunology, Johns Hopkins University Bloomberg School of Public Health, Baltimore, MD, USA; 6 Department of Biochemistry and Molecular Biology, Johns Hopkins University Bloomberg School of Public Health, Baltimore, MD, USA

**Keywords:** Human Papillomavirus 16, E7 antigen, E6 antigen, DNA vaccine, cell-penetrating peptide, signal sequence, immunotherapy

## Abstract

**Background:**

Cervical cancer is the fourth most common cancer affecting women, with almost all cases caused by infection with Human Papillomavirus (HPV). Previous research has explored the potential of cell-penetrating peptides to improve the efficacy of peptide vaccines and signal peptide sequences to increase protein secretion in DNA vaccines. In this study, we evaluated the in vivo efficacy of DNA vaccines targeting HPV-16 E6 and E7 viral antigens that are constructed with both a cell-penetrating peptide and IL-2 signal peptide to improve immunogenicity.

**Results:**

We generated DNA vaccine constructs utilizing combinations of E7(49–57), E6(48–57), IL-2 signal sequence (sig), 9-polyarginine (9R), and 12-polyarginine (12R) cloned into pcDNA3 vectors. Transfection of the pcDNA3-sig9RE7, pcDNA3-sig12RE7, or pcDNA3–9RE7 vaccine constructs induced a significant E7-specific CD8+ T cell response in vitro. We observed that the pcDNA3-sig9RE7 vaccine induced the strongest E7-specific immune response in vivo compared to the pcDNA3–9RE7 and pcDNA3-sig12RE7 constructs. We demonstrated the potential protective and therapeutic anti-tumor effects of the pcDNA3-sig9RE7 vaccination against the HPV-16 E7 expressing TC-1 tumor model in mice. Finally, we found similar anti-tumor effects for the pcDNA3-sig9RE6 vaccine which suggest this DNA vaccine strategy could be applied to various cervical cancer antigens.

**Conclusion:**

Treatment with the pcDNA3-sig9RE7 DNA vaccine induces a potent E7-specific anti-tumor immune response in mice. By demonstrating the potential of utilizing a cell-penetrating peptide and signal peptide sequence in combination to improve efficacy, this study provides a foundation for future research into fusion DNA vaccines with cell-penetrating peptides and signal peptide combinations.

## Introduction:

Worldwide, cervical cancer is the fourth most common cancer affecting women [1,2]. Almost all cases of cervical cancer are caused by infection with high-risk types of Human Papillomavirus (HPV), including HPV-16 and HPV-18 which cause over 70% of cervical cancers [1–3]. Current vaccines are effective prophylactically to prevent HPV infection but cannot treat existing infections [2,4,5]. Because of these challenges, much research has been done to find potential treatments for ongoing HPV infections and its resulting malignancies.

HPV causes the production of the viral proteins E6 and E7, which are the main drivers of infected cell transformation [2]. E6 and E7 have been shown to increase infected cell transformation and inhibit tumor suppressor genes [6]. Because these proteins are foreign, they are only expressed in infected cells and can be identified as tumor-specific antigens [2,7]. This makes E6 and E7 ideal targets for immunotherapies to treat HPV-associated cancers [2,7]. Most therapeutic vaccines target E7 as it is better characterized and the associated immune response has been more deeply studied [2,8]. However, there is still a need to further evaluate E6 as it is highly expressed in HPV-16 infected cells and studies have found that E6 targeting vaccines can control E6-expressing tumors [8].

Due to the lack of effective treatments for existing HPV infections, significant research has been done to study potential vaccines to treat HPV cervical cancers. Therapeutic HPV vaccines aim to utilize the immune system by inducing T cell-mediated immune responses that target HPV infected cells. Peptide vaccines have been studied as one approach to HPV treatment. These vaccines function by priming the patient’s T cells with exposure to the tumor antigen but face challenges in creating a potent response [9]. Because of this, many peptide vaccines include additional elements such as cell-penetrating peptides (CPPs) to improve efficacy [9]. This strategy aims to improve the delivery of the vaccine molecule itself.

For peptide vaccines to be effective, the peptide antigens should be trafficked to the lymph nodes (LNs) and taken up by antigen presenting cells (APCs). CPPs have been shown to increase peptide antigen trafficking from the vaccination site to the draining LNs (dLNs) [9]. One proposed method of action is through the interactions of CPPs with serum proteins in the blood, mainly Apolipoprotein A [19,10]. The use of CPPs in peptide vaccines allows for increased antigen trafficking and extended antigen presentation in the dLNs [9,11]. CPPs are generally less than 30 amino acids long and include many positively charged residues to promote effective permeation of the cell membrane [10,12,13]. Therefore, polyarginine is a common cell-penetrating peptide that we are utilizing due to its strong positive charge and simple construction [10,12,13].

An additional approach to HPV therapeutic treatments is the DNA vaccine, which is useful because of its stability and low cost of production [2]. However, DNA vaccines cannot amplify to other cells in vivo and this results in a low immunogenicity. There have been many attempts to increase DNA vaccine efficacy, including the fusion of signal peptide sequences. DNA vaccines created with signal peptide sequences have been shown to cause increased protein secretion [14,15]. This ensures the DNA vaccine is translated and secreted from the cell, where it can be trafficked to the lymph nodes. A signal peptide sequence used in other studies is the Interleukin-2 (IL-2) signal peptide because it leads to the secretion of IL-2 [14,15].

Therefore, this study aimed to determine the potential of a DNA vaccine targeting the HPV-16 E6 or E7 viral antigen that was constructed with both a CPP and IL-2 signal peptide to improve immunogenicity. Furthermore, our study evaluated the in vivo efficacy of this fusion construct in generating anti-tumor immune response against HPV-16 E7 TC-1 tumor cells in mice. Finally, we tested the in vivo efficacy of a vaccine targeting HPV-16 E6 to determine whether this fusion construct is applicable to various cervical cancer antigens.

## Methods

### Mice

Six to eight-week-old C557BL/6 mice were purchased from the National Cancer Institute (Frederick, Maryland, USA) and kept in the oncology animal facilities at Johns Hopkins Hospital (Baltimore, Maryland, USA). All animal procedures were done following the Johns Hopkins Institutional Animal Care and Use Committee protocols and in accordance with recommendations for the proper care of lab animals.

### Cell lines

The HPV16-E6/7 expressing TC-1 cell line has been previously described [16]. TC-1 cells were cultured in RPMI-1640 medium supplemented with 2 mM glutamine, 1 mM sodium pyruvate, 100 U/mL penicillin, 100 μg/mL streptomycin, 5 × 10–5 M β-mercaptoethanol, and 10% fetal bovine serum.

The 293-Db cell line has been previously described [17]. 293-Db cells were cultured in DMEM medium supplemented with 2 mM glutamine, 1 mM sodium pyruvate, 100 U/mL penicillin, 100 μg/mL streptomycin, and 10% fetal bovine serum.

The murine E7aa49–57 peptide specific CD8+ T cell line has been previously described [18]. This E7-specific CD8+ T cell line were cultured at 37 °C with 5% CO2 in RPMI-1640 medium supplemented with 10% FBS, 2 mM l-glutamine, 1 mM sodium pyruvate, 2 mM non-essential amino acids, 100 U/mL penicillin, 100 μg/mL streptomycin, 55 μM 2-Mercaptoethanol, and 25 IU/mL IL-2.

### DNA constructs

The generation of pcDNA3-E7(49–57) (PMID 28852471) has been described previously. Constructs including pcDNA3-sigE7, pcDNA3–9RE7, pcDNA3-sig9RE7, pcDNA3-sig12RE7, pcDNA3-E6, and pcDNA3-sig9RE6 were synthesized by GenScript Corporation and cloned into pcDNA3.1(−). The corresponding amino acid sequences are listed in Table 1.

### In vitro T cell activation assay

293-Db cells were transfected with pcDNA3, pcDNA3-E7, pcDNA3-sigE7, pcDNA3–9RE7, pcDNA3-sig9RE7, or pcDNA3-sig12RE7 using Lipofectamine 2000. 24 hours after transfection, the cells were incubated with HPV16-E7aa49–57 peptide specific CD8+ T cell line at an E:T ratio of 1 in the presence of GolgiPlug (1 μL/mL; BD Pharmingen, San Diego, CA) for 20 h at 37 °C. 293-Db cells without treatment were used as a negative control while cells loaded with HPV16-E7 aa49–57 peptide were used as a positive control. After incubation, the CD8+ T cells were collected, washed with FACS wash buffer (PBS containing 0.5% BSA), stained with phycoerythrin-conjugated monoclonal rat anti-mouse CD8 antibody (BD Pharmingen, San Diego, CA). The cells were then fixed using the Cytofix/Cytoperm kit (BD Pharmingen, San Diego, CA), and intracellularly stained with FITC-conjugated anti-mouse IFN-γ antibody (BD Pharmingen, San Diego, CA). After washing, the cells were acquired with the FACSCalibur flow cytometer and analyzed by CELLQuest Pro software (BD Bioscience, Mountain View, CA).

### Electroporation-mediated DNA vaccination

For an intramuscular (IM) vaccination with electroporation, either 10 μg of E7, sigE7, 9RE7, sig9RE7, or sig12RE7, or sig9RE7 vectors were prepared and injected in the tibialis muscle of the shaved hind leg of mice followed by electroporation with an ECM830 Square Wave Electroporation System (BTX Harvard Apparatus company, Holliston, MA, USA). Booster vaccinations were administered using the same dose and regimen as above.

### Intracellular cytokine staining with flow cytometric analysis to detect IFN-γ secretion by E7-specific CD8+ T cells

Splenocytes from vaccinated mice were collected and incubated for 20 hours with 1 μg/mL of MHC class I restricted E7 peptide (aa49–57, RAHYNIVTF). 1 μL/mL of GolgiPlug was then added 6 hours before the cells were harvested. After incubation, the cells were washed with FACScan buffer and then stained with PE-conjugated monoclonal rat anti-mouse CD8 antibody. The cells were then fixed using the Cytofix/Cytoperm kit. Intracellular IFN- γ was stained with FITC-conjugated anti-mouse IFN-γ antibody. After staining and washing, the cells were acquired with the FACSCalibur flow cytometer and analyzed by CELLQuest Pro software.

### In vivo tumor protection

C57BL/6 mice (5 per group) were vaccinated with either 10 μg of pcDNA3-E7(49–57), pcDNA3-sig9RE7, pcDNA3-E6, or pcDNA-sig9RE6 via IM injection with electroporation followed by a booster vaccination 1 week later. A week after the last vaccination, mice were challenged subcutaneously (SC) with 1×10^5 TC-1 cells/mice. Mice challenged with TC-1 cells without treatment were used as controls. For 100 days after tumor challenge, mouse survival and tumor growth by direct palpation were measured. When their tumor growth exceeded 1.5 cm in diameter, the mice were considered to have died from tumor burden and were subsequently euthanized.

### In vivo tumor treatment

C57BL/6 mice (5 per group) were challenged SC with 1×10^5 TC-1 cells/mouse. Four days after tumor challenge, mice (5 per group) were vaccinated with either 10 μg of pcDNA3-E7(49–57), pcDNA3-sig9RE7, pcDNA3-E6, or pcDNA3-sig9RE6 via IM injection with electroporation. This was followed by booster vaccinations on days 10 and 16. Tumor challenged mice without vaccinations were used as controls. Once a week, tumor growth was determined by direct palpation, and the formation of a detectable tumor was noted. When the tumor growth exceeds 1.5 cm in diameter, the mice were considered to have died from tumor burden and were subsequently euthanized.

### Statistical analysis

Data expressed as mean ± standard deviation represent a minimum of two different experiments. Two-tailed student’s t tests were used to make comparisons between individual data points. Survival distributions for different in vivo groups were compared through Kaplan-Meier curves and Log-rank tests. A p < 0.05 was considered statistically significant.

## Results

Presentation of HPV16-E7 by 293-Db cells transfected with pcDNA3-sig9RE7

**Figure 1:**
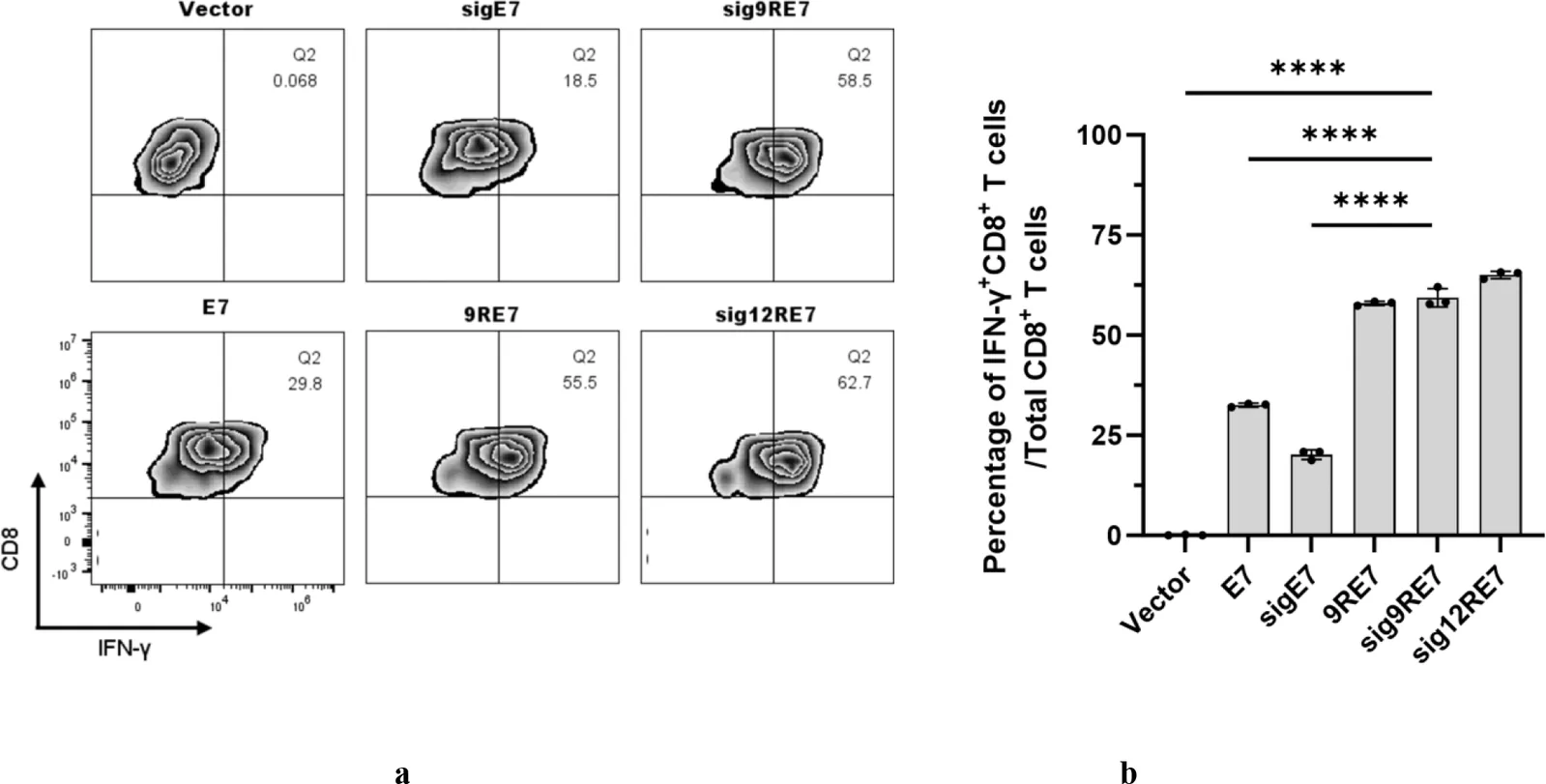
Presentation of the HPV16-E7 antigen by 293-Db cells transfected with various DNA vaccine constructs. Transfected 293-Db cells were cocultured with a HPV16-E7-specific CD8+ T cell line (E:T ratio of 1:1). After co-incubation, the E7-specific CD8+ T cells were stained for surface CD8 and intracellular IFN- γ and acquired with a flow cytometer. **a** Flow cytometry plots illustrate the number of E7-specific IFN- γ CD8+ T cells. **b** Bar graph summary of the flow cytometry results.

To evaluate the E7 antigen presentation, we transfected 293-Db cells with various constructs. The constructs used were pcDNA3, pcDNA3-E7, pcDNA3-sigE7, pcDNA3–9RE7, pcDNA3-sig9RE7, and pcDNA3-sig12RE7. After transfection, the 293-Db cells were cocultured with an HPV-16 E7-specific CD8+ T cell line at an effector-to-target ratio of 1:1. The E7-specific CD8+ T cells were then stained for surface CD8 and intracellular IFN- γ and analyzed using flow cytometry. As shown in Fig. 1, 293-Db cells transfected with the pcDNA3-sig9RE7, pcDNA3-sig12RE7, or pcDNA3–9RE7 constructs generated the three strongest E7-specific CD8+ T cell responses. The pcDNA3-E7 and pcDNA3-sigE7 constructs also showed significant E7-specific CD8+ T cell responses while the empty vector construct showed a negligible response (Fig. 1).

### PcDNA3-sig9RE7 vaccine induced potent E7-specific immune response in vivo

**Figure 2:**
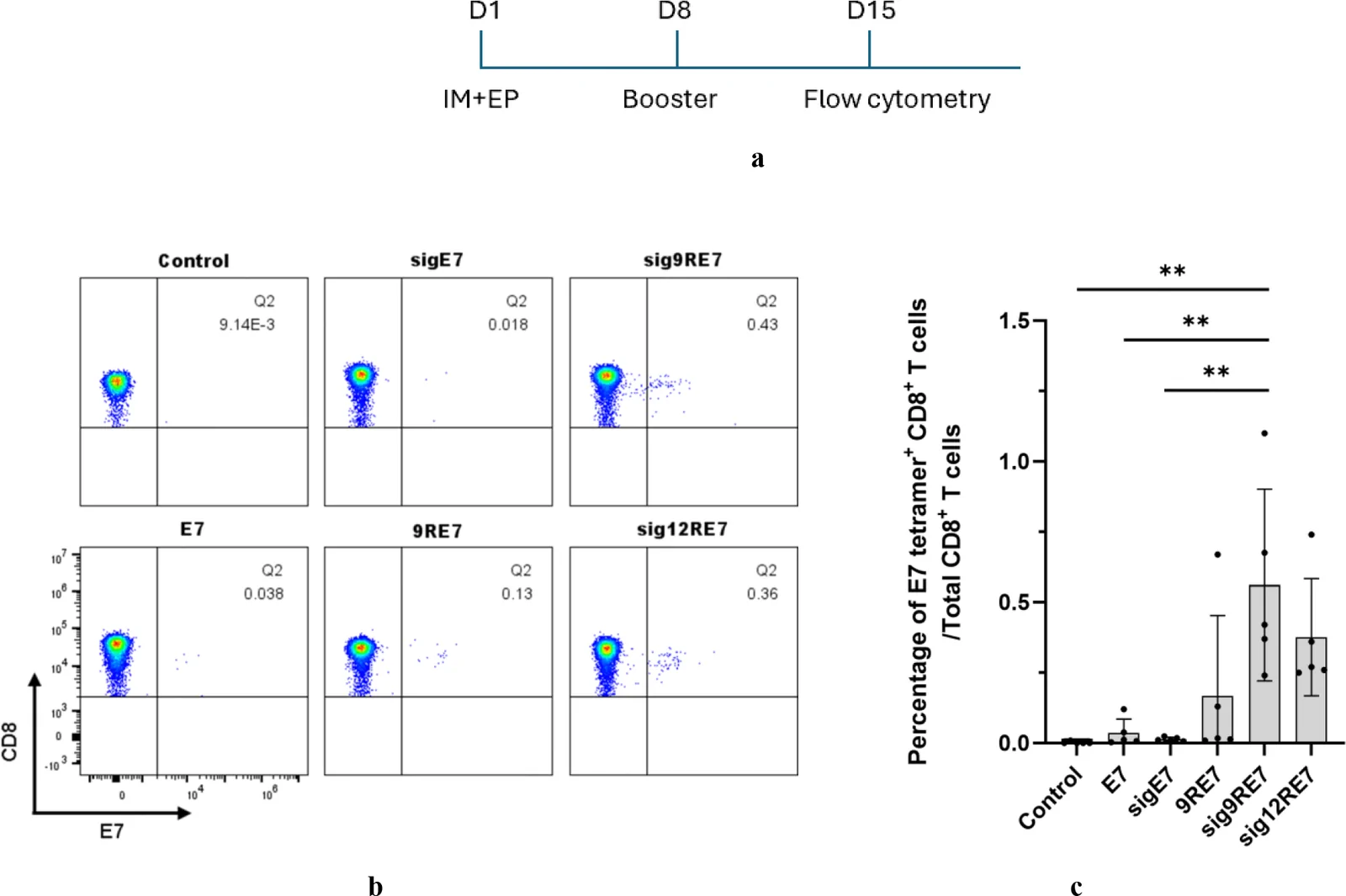
Analysis of the in vivo E7-specific immune response generated by various DNA vaccines. **a** Briefly, 6–8-week-old female C57BL/6 mice were immunized IM with 10 μg of pcDNA3-E7, pcDNA3-sigE7, pcDNA3–9RE7, pcDNA3-sig9RE7, pcDNA3-sig12RE7, or pcDNA3-sig9RE7 vectors followed by electroporation on day 1. The mice were boosted with the same regimen on day 8. **b c** Seven days after the booster, PBMCs were collected and the lymphocytes were stained with a surface CD8 antibody and an E7 tetramer, then analyzed by flow cytometry. **b** Representative flow cytometry plots of the frequency of E7-specific CD8+ T cells within the CD8+ T cell population. **c** Bar graph summarizes the flow cytometry analysis.

The immunogenicity of E7 antigen-specific constructs were evaluated in vivo by vaccinating mice IM in their hind legs with either the control, E7, sigE7, 9RE7, sig9RE7, or sig12RE7, or sig9RE7 vectors. The injection was followed by electroporation. This same regimen was administered again one week later. One week after the booster immunization, peripheral blood mononuclear cells (PBMCs) were collected to assess the immune response. The lymphocytes were stained with a surface CD8 antibody and an E7 tetramer, then analyzed by flow cytometry. The mice vaccinated with the control, E7, or sigE7 vectors showed a very weak E7-specific CD8+ T cell response (Fig. 2b,c). In contrast, the mice treated with the sig9RE7 vector showed the most robust E7-specific CD8+ T cell response (Fig. 2b,c). The mice treated with sig12RE7 or 9RE7 vectors also induced a robust E7-specific CD8+ T cell response (Fig. 2b,c).

After determining that the sig9RE7 vaccine had the strongest E7-specific immune response, we chose to continue evaluating the potential of only this construct against E7 or control vaccines. Continuing to evaluate the sig12RE7, sigE7, and 9Re7 constructs was unnecessary in evaluating the potential of utilizing a CPP and signal sequence fusion vaccine construct. This choice simultaneously reduces the burden of our experiments by utilizing less mice and resources while evaluating the DNA vaccine constructs that are essential for determining proof-of-concept.

### PcDNA3-sig9RE7 vaccination induced potent protective effects against tumor challenge in vivo

**Figure 3:**
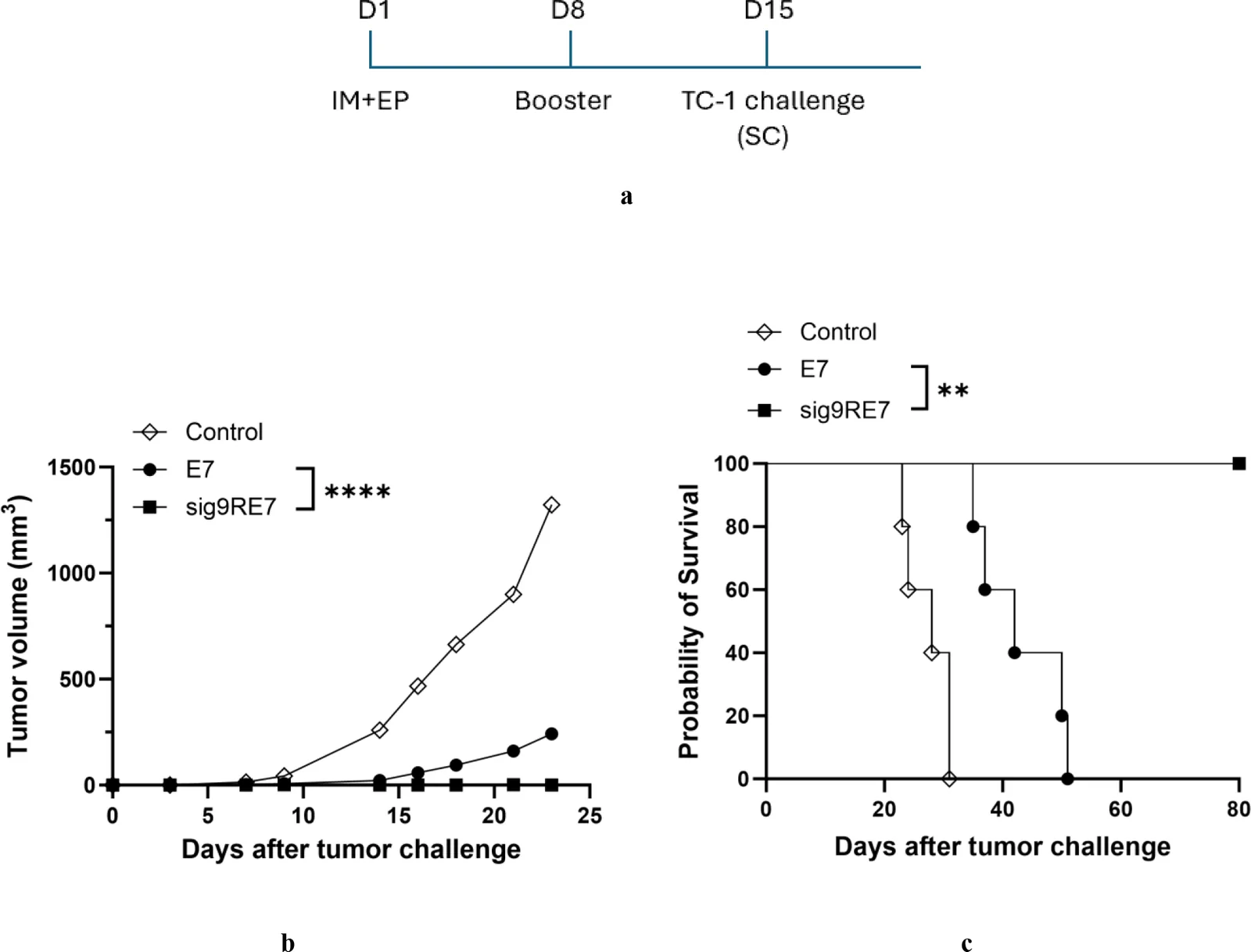
Prophylactic pcDNA3-sig9RE7 vaccination induced protective anti-tumor effects in vivo. **a** Briefly, 6–8-week-old female C57BL/6 mice were vaccinated IM with 10 μg of pcDNA3-E7 or pcDNA3-sig9RE7 vectors followed by electroporation on day 1. The mice were boosted with the same regimen on day 8. Untreated mice were used as a negative control. Seven days after the last vaccination, mice were challenged subcutaneously with 1 × 10^5 TC-1 tumor cells, and tumor size and survival were monitored over time. **b** Line graph depicting the change in tumor volume in TC-1 challenged mice over 25 days. **c** Kaplan-Meier analysis was done to evaluate and plot the survival rates of mice bearing TC-1 tumors.

To evaluate whether pcDNA3-sig9RE7 vaccination can generate antitumor effect against a tumor challenge, mice were immunized by the same regimen as shown in Fig. 2. A week after the last vaccination, mice were challenged SC with TC-1 tumor cells. Unvaccinated TC-1 challenged mice were used as controls. After the tumor challenge, the mice were monitored for tumor size and survival over time. No observable tumor growth was observed in the mice vaccinated with pcDNA3-sig9RE7 compared to other treatment groups (Fig. 3b). Additionally, all mice treated with pcDNA3-sigRE7 survived over 80 days after the tumor challenge while all mice in the other treatment groups died within 60 days after the tumor challenge (Fig. 3c). This data suggests that the pcDNA3-sig9RE7 vaccine can provide protection against TC-1 tumor challenges by generating a potent immune response.

### Vaccination by pcDNA3-sig9RE7 generated potent antitumor effects in TC-1 tumor challenged mice

**Figure 4:**
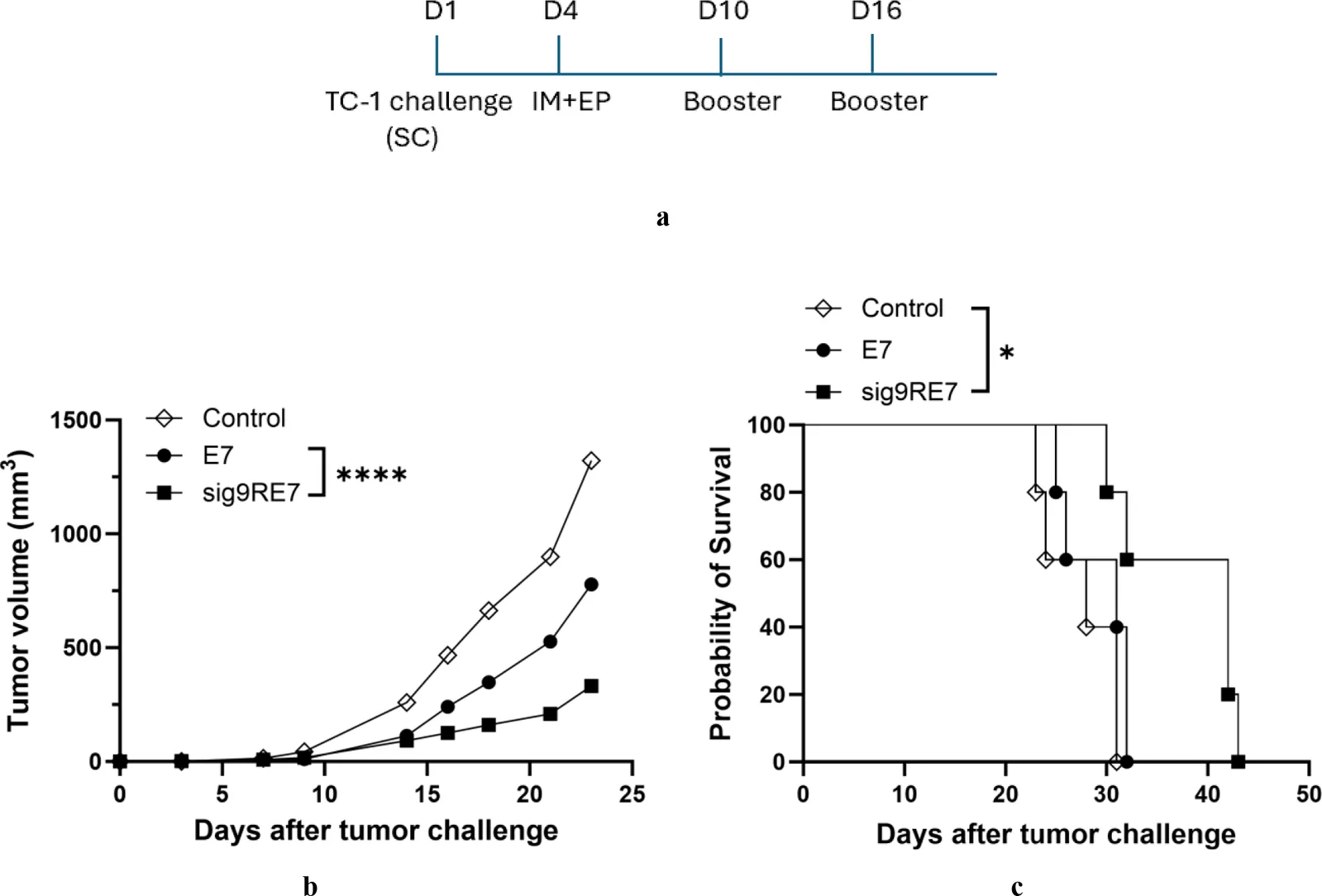
Vaccination with the pcDNA3-sig9RE7 construct induced potent anti-tumor effects against TC-1 tumor cells. a Briefly, female C57BL/6 mice 6–8 weeks old were challenged SC with 1×10^5 TC-1 cells. Four days after tumor challenge, mice were vaccinated with either 10 μg of pcDNA3-E7(49–57) or pcDNA3-sig9RE7 via IM injection with electroporation. Mice were boosted with the same regimen on days 10 and 16. Tumor-challenged mice without vaccinations were used as controls and tumor growth and survival were monitored over time. b Line graph showing the change in tumor volume of TC-1 challenged mice over 25 days. c Kaplan-Meier survival plot of tumor-bearing mice was recorded over 50 days.

After demonstrating that the pcDNA3-sig9RE7 can protect mice from TC-1 tumor challenge when administered prophylactically, we aimed to determine whether the vaccine could be used to treat mice already bearing TC-1 tumors. Mice were challenged with TC-1 tumor cells SC on day 1. Mice then received a vaccination IM of pcDNA3-sig9RE7 or pcDNA3-E7 on day 4 and boosted with the same regimen on day 10 and day 16. Mice in the control group received no DNA vaccination. After the booster, mice were monitored for tumor growth and survival over time. Mice treated with the pcDNA3-sig9RE7 vaccine had the smallest tumors and longest survival time of over 40 days (Fig. 4b). Mice treated with pcDNA3-E7 and those in the control group showed larger tumor volumes and shorter survival times of less than 35 days (Fig. 4c). This indicates a potential for the pcDNA3-sig9RE7 vaccine to be used therapeutically to control the growth of tumors.

### Vaccination by pcDNA3-sig9RE6 induced potent protective effects against tumor challenge in vivo

**Figure 5:**
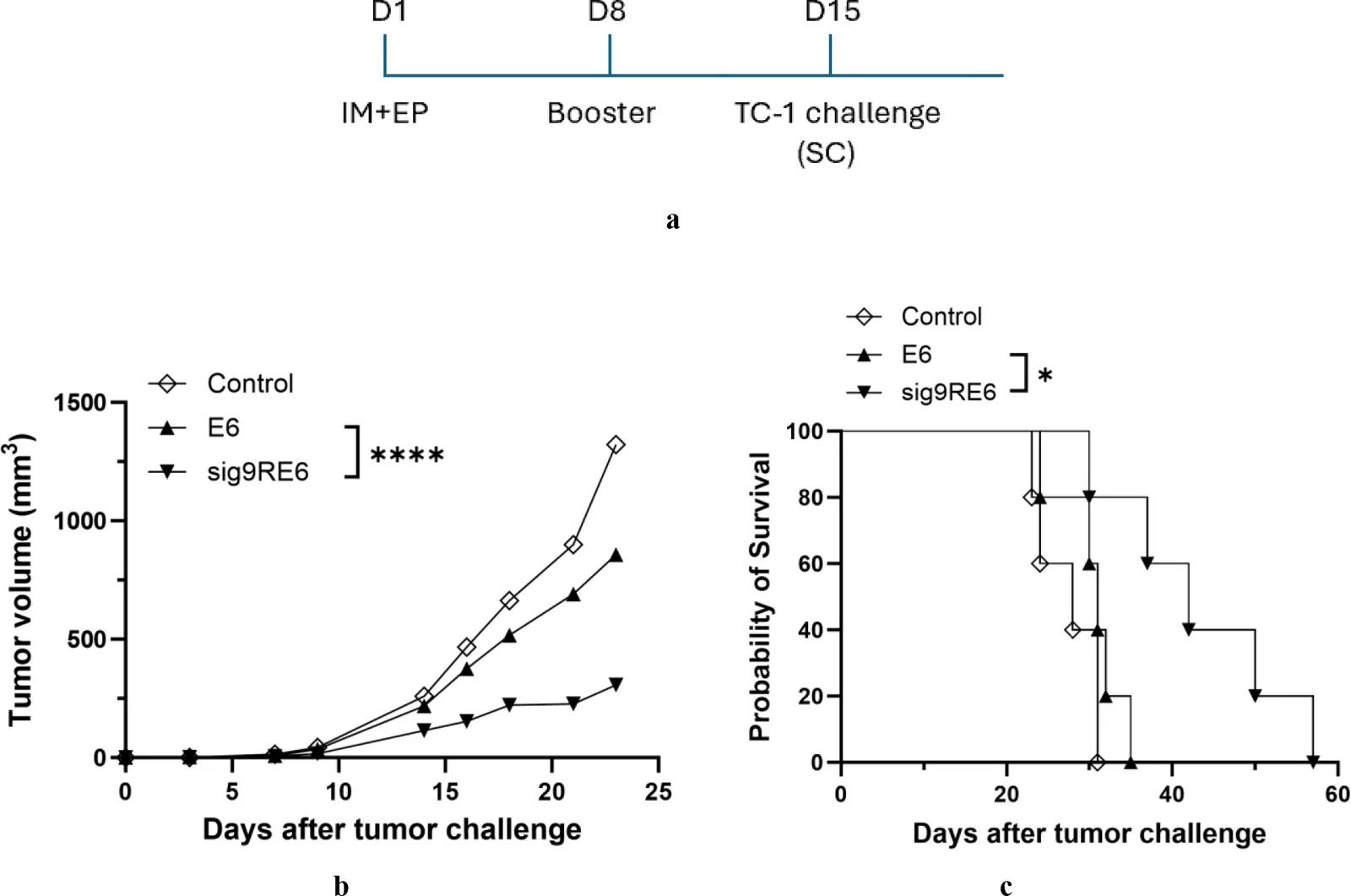
Prophylactic pcDNA3-sig9RE6 vaccination induces protective anti-tumor effects in mice. a Briefly, 6–8-week-old female C57BL/6 mice were vaccinated IM with 10 μg of pcDNA3-E6 or pcDNA-3sig9RE6 vectors followed by electroporation on day 1. The mice were boosted with the same regimen on day 8. Untreated mice were used as a negative control. Seven days after the last vaccination, mice were challenged subcutaneously with 1 × 10^5 TC-1 tumor cells, and tumor size and survival were monitored over time. b Line plot depicting tumor volume of TC-1 challenged mice over 25 days. c Kaplan-Meier survival plot of tumor-bearing mice was recorded over 60 days.

After determining the potential of using the pcDNA3-sig9RE7 vaccine against tumors, we aimed to evaluate whether this effect extends to other antigens present in cervical cancer. To test whether pcDNA3-sig9RE6 could also generate a protective anti-tumor effect against tumor challenges, mice were vaccinated with the same regimen as shown in Fig. 2. One week after the booster vaccine, mice were challenged SC with TC-1 tumor cells. After the tumor challenge, mice were monitored for tumor size and survival over time. Mice vaccinated with the pcDNA3-sig9RE6 demonstrated the smallest amount of tumor growth when compared with the pcDNA3-E6 and control groups (Fig. 5b). Furthermore, mice treated with pcDNA3-sig9RE6 survived for over 55 days while the pcDNA3-E6 and control groups had a lower survival of under 40 days (Fig. 5c). This data suggests that the pcDNA3-sig9RE6 vaccine can provide protection against TC-1 tumor challenges.

### pcDNA3-sig9RE6 immunization generated antitumor effects in tumor challenged mice

**Figure 6:**
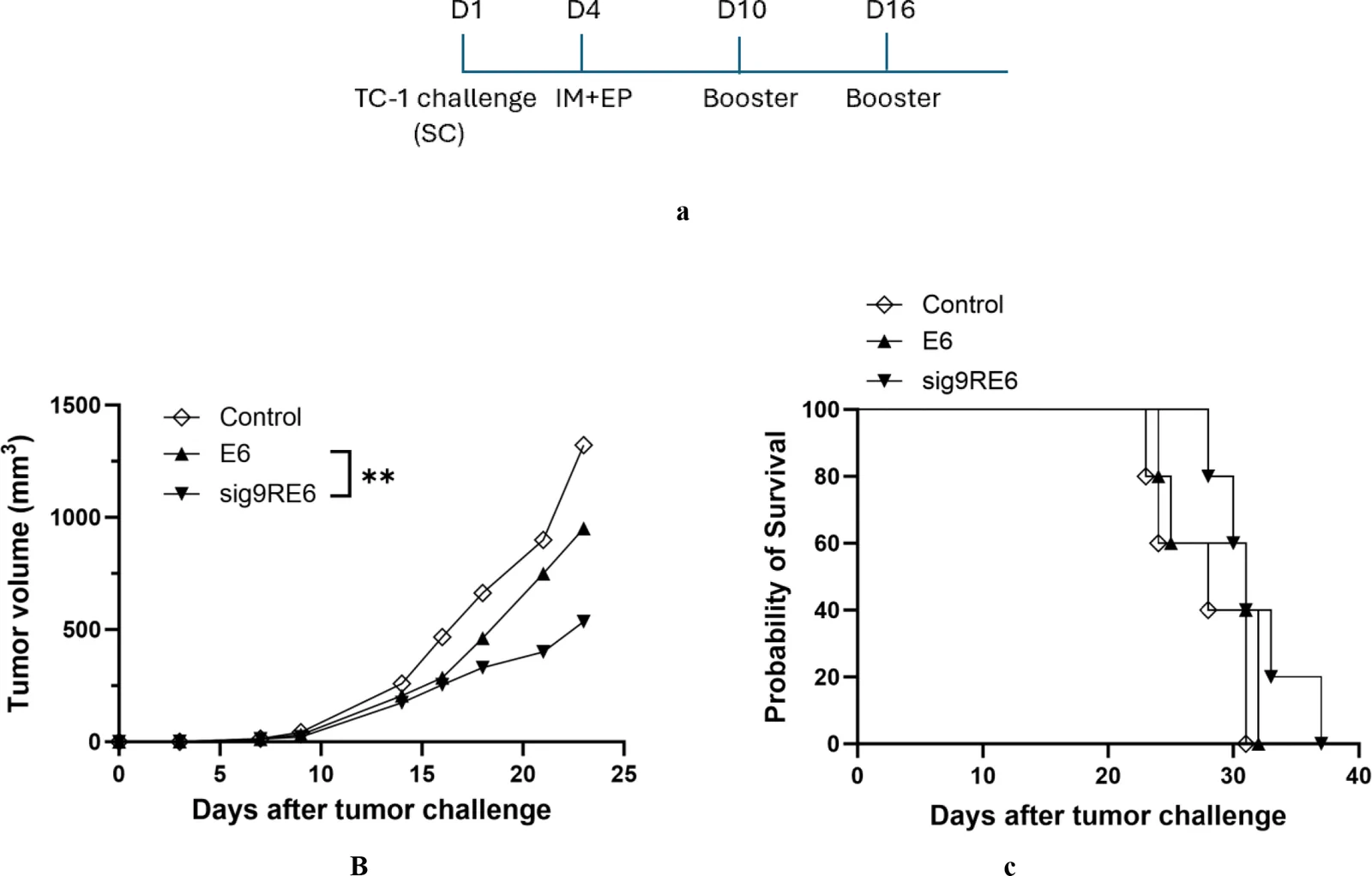
Vaccination with pcDNA3-sig9RE6 induced anti-tumor effects in tumor challenged mice. **a** Briefly, female C57BL/6 mice 6–8 weeks old were challenged SC with 1×10^5 TC-1 cells. Four days after tumor challenge, mice were vaccinated with either 10 μg of pcDNA3-E6(48–57) or pcDNA3-sig9RE6 via IM injection with electroporation. Mice were boosted with the same regimen on days 10 and 16. Tumor-challenged mice without vaccinations were used as a control, and tumor growth and survival were monitored over time. **b** Line graph depicting the change in tumor volume of TC-1 challenged mice over 25 days. c Kaplan-Meier survival plot of tumor-bearing mice was recorded over 40 days.

After determining the prophylactic potential of the pcDNA3-sig9RE6 vaccine, we aimed to evaluate the potential of the pcDNA3-sig9RE6 vaccination to treat mice already burdened with tumors. Mice were challenged with TC-1 cells and immunized following the same regimen as in Figure 4. Mice received vaccinations with either pcDNA3-sig9RE6 or pcDNA3-E6, with control mice receiving no vaccination. After immunization, the mice were observed for tumor size and survival over time. Mice treated with the pcDNA3-sig9RE6 vaccine showed the smallest amount of tumor growth when compared with the pcDNA3-E6 and control mice (Fig. 6b). Additionally, mice vaccinated with pcDNA3-sig9RE6 had the longest survival time of over 35 days (Fig. 6c). Mice in the pcDNA3-E6 and control groups survived for less than 35 days (Fig. 6c). This data indicates a potential for the pcDNA3-sig9RE6 vaccine for controlling the growth of tumors.

## Discussion

In this study, we determined that the pcDNA3-sig9RE7 vaccine induced a stronger E7-specific CD8+ T cell-mediated immune response than the pcDNA3-E7 vaccine. We also demonstrated the potential protective and therapeutic anti-tumor effects of the pcDNA3-sig9RE7 vaccination against the HPV-16 E7 expressing TC-1 tumor model in mice. Finally, we found similar anti-tumor effects for the pcDNA3-sig9RE6 vaccine which suggest this DNA vaccine strategy could be applied to various cervical cancer antigens. Previous research has explored the increased immunogenicity of peptide vaccines that utilize cell-penetrating peptides and DNA vaccines that use signal peptide sequences. However, this study is the first time a CPP and signal sequence were utilized together in a DNA vaccine treatment to increase efficacy.

Our study demonstrated the potential significance of a cationic CPP in inducing the CD8+ T cell response in vivo. We observed an increase in the CD8+ T cell response in vivo after treatment with the pcDNA3–9RE7 construct as well as the constructs with both a CPP and signal sequence. This indicates that the CPP itself contributes to the immunogenicity of the vaccine. These results contrast with previous studies that evaluated the use of HIV trans-activator of transcription (Tat) as a CPP for multiple-epitope DNA vaccines [19]. Their results showed no significant increase in immunogenicity or CD8+ T cell responses in vivo with the use of HIV-Tat compared to the vaccines without a CPP. The significant increase in immunogenicity from our vaccine could be caused by utilizing polyarginine as opposed to HIV-Tat. Because their mechanisms of action are not fully understood, these arginine-rich CPPs likely have different properties or interactions that affect their immunogenicity.

In determining the immunogenicity of the 9R and 12R fusion vaccines, we simultaneously evaluated whether the length of the polyarginine sequence holds significance in vaccine efficacy. Our results demonstrate the increased functionality of 9R compared to 12R in the DNA vaccine constructs. We showed that 9R in combination with the signal sequence and E7 antigen creates a more robust CD8+ T cell response compared with the 12R signal peptide fusion construct.

Our study did not investigate the mechanism of action for the increased immunogenicity of our polyarginine signal sequence fusion constructs. Because CPPs can be created from many different protein structures, they have diverse mechanisms of membrane penetration that are difficult to predict from their structure alone [12,13,20,21]. Attempts have been made to evaluate the mechanisms of action for CPPs, but many are still not fully understood. Besides increasing membrane penetration, research has suggested that antigen peptide-CPP vaccines rely on the cross-presentation by specialized dendritic cells to generate an effective immune response [9,11]. Many CPPs were shown to interact with serum proteins, which increased lymph trafficking and therefore generated a stronger immune response in vivo [9,10]. As these studies only evaluated a few CPPs out of many, there are opportunities for further research into a more diverse set of CPPs.

By evaluating the potential of the HPV-16 viral antigens E6 and E7 as target antigens for the DNA vaccine, we found that the E7 antigen was a more potent target. Increased vaccine efficacy was demonstrated by the longer survival times of sig9RE7 vaccinated mice compared to sig9RE6 mice when challenged with tumor cells. However, this study did not explore the potential of utilizing a DNA vaccine constructed with E6 and E7 antigens in combination. Research has demonstrated that multiple epitope DNA vaccines are effective in targeting multiple antigens without losing potency [19,22]. Thus, more research is needed to determine whether a vaccine targeting both E7 and E6 simultaneously would increase efficacy compared to single epitope vaccines.

Another potential method to increase vaccine potency is to alter the construction of the vaccine structure itself by using different amino acid linkers. There has been research into both furin sensitive and furin resistant linkers as possible additions to peptide vaccines [19,23]. A study by Lu et al. determined that furin sensitive linkers in Trojan antigen targeting vaccines played a role in sensitizing target cells for T cell lysis. However, another study found that furin sensitive linkers have no significant effect on the immunogenicity of multi-epitope peptide vaccines [19]. These results warrant further research into the effects of various linker sequences on vaccine efficacy.

While most investigations into CPP efficacy have been performed using peptide vaccines, we chose to utilize DNA vaccinations because of their stability and ease of use [2,9]. These vaccine types utilize different mechanisms of action as DNA vaccines require cellular uptake of the DNA vector and protein secretion while peptide vaccines directly provide the target antigens. Further evaluation of various CPPs in DNA vaccines is therefore necessary to determine their efficacy. Utilizing CPP-signal sequence fusion constructs could also be tested in other modalities such as a therapeutic mRNA vaccine in the future.

The possibility that the HPV-16 E7 fusion vaccine can induce an E7-specific CD8+ T cell response and create therapeutic anti-tumor effects in vivo holds significant translational value. The pcDNA3-sig9RE7 fusion construct serves as proof-of-concept for the construction of a therapeutic DNA vaccine targeting HPV cervical cancer. This vaccination would only serve as one part of a combination therapy to treat cervical cancer and should be combined with other treatments such as chemotherapy and radiation to provide the best treatment [24]. While it is likely that more effective CPP and signal sequence combinations will be discovered, our results provide a foundation for future research and demonstrate the translational potential of DNA fusion vaccines to treat cervical cancer.

## Conclusion

In summary, this study demonstrates the potential of utilizing a cell-penetrating peptide and signal peptide sequence in combination to improve the anti-tumor immunogenicity of a DNA vaccine to treat HPV cervical cancer. Alongside significant research by others to discover effective CPPs and signal peptide sequences, this study provides a foundation for future research into fusion DNA vaccines with more immunogenic CPP and signal peptide sequence combinations.

## Figures and Tables

**Table 1:** Table of DNA constructs and corresponding amino acid sequences. DNA constructs include combinations of E7(49–57), E6(48–57), IL-2 signal peptide, 9R, 12R that were synthesized and cloned into the pcDNA3.1 vector.

DNA Construct	Amino Acid Sequence
pcDNA3-E7	RAHYNIVTF
pcDNA-sigE7	MQLLSCIALSLALYTNS - RAHYNIVTF
pcDNA3-9RE7	RRRRRRRRR - RAHYNIVTF
pcDNA3-sig9RE7	MQLLSCIALSLALYTNS – RRRRRRRRR – RAHYNIVTF
pcDNA3-sig12RE7	MQLLSCIALSLALYTNS – RRRRRRRRRRRR – RAHYNIVTF
pcDNA-E6	EVYDFAFRDL
pcDNA-sig9RE6	MQLLSCIALSLALYTNS – RRRRRRRRR – EVYDFAFRDL
